# Novel model for load carriage ergonomics optimisation

**DOI:** 10.1186/2046-7648-4-S1-A12

**Published:** 2015-09-14

**Authors:** Amir Hadid, Noa Belzer, Amit Gefen, Nogah Shabshin, Yoram Epstein

**Affiliations:** 1 Department of Biomedical Engineering, Tel Aviv University, Division of Diagnostic Imaging, Carmel Medical Center, Heller Institute of Medical Research, Sheba Medical Center, Sackler Faculty of Medicine, Tel Aviv University, Israel

## Introduction

Soldiers and recreational backpackers are often required to carry heavy loads during military operations or hiking. Despite the advances in backpack design, the loads carried by soldiers still impose an extreme physiological strain (soft tissue deformation) which frequently results in discomfort, pain, and musculoskeletal injuries. Shoulder strain, which appears to be one of the limiting factors of load carriage, is thought to result from higher susceptibility to short-term injuries such as soft tissue damage and/or trapped nerves and obstruction of blood vessels. The aim of the current study was to develop a flexible model enabling to simulate real life loading scenarios (various loads, strap materials and strap structures) and that will help in optimising load carriage systems design and guidelines.

## Methods

Open-MRI scans were used for reconstructing a 3D geometrical model of an unloaded shoulder and for measuring the soft tissue deformations caused by a 25 kg backpack; subsequently, a subject-specific finite element (FE) stress-strain analyses was developed. In this model, loads were applied at the strap-shoulder contact surfaces by pulling the strap towards the shoulder until the desired load was reached. Then, the model enables to calculate and analyse the strains in the soft tissues surrounding the brachial plexus.

## Results

The newly developed model successfully enabled the prediction of soft tissue deformations in the brachial plexus surrounding tissues, which are caused by different backpack loads. Increasing the loads up to 35 kg (or ~45% of a typical bodyweight) resulted in further increase in strains of the underlying soft tissues: the maximal tensile strain in the brachial plexus for a 25 kg backpack was 12% and while carrying 35 kg the maximal tensile strain increased to 16%. It also had been found in the study that the lateral aspects of the brachial plexus are more vulnerable to a deformation-induced injury, because at these aspects the nerve plexus is less protected against the compressive loads applied during load carriage.

## Discussion

In addition, the model allows a better understanding of the more susceptible anatomical regions in the backpack-wearer interface. Based on previous animal studies, the soft tissue strains in the area of the brachial plexus as calculated by the model are substantial and might hamper normal nerve conductivity.

## Conclusion

This method is potentially a strong working tool allowing further developments of new strap structures and materials or backpack design for alleviating the strains applied on the shoulder's soft tissues.

